# A Comprehensive Review of the Cardiovascular Protective Properties of Silibinin/Silymarin: A New Kid on the Block

**DOI:** 10.3390/ph15050538

**Published:** 2022-04-27

**Authors:** Nikolaos P. E. Kadoglou, Chrystalla Panayiotou, Michail Vardas, Nikolaos Balaskas, Nikolaos G. Kostomitsopoulos, Alexandra K. Tsaroucha, Georgia Valsami

**Affiliations:** 1 Medical School, University of Cyprus, Nicosia 2109, Cyprus; panayiotou.e.chrystalla@ucy.ac.cy (C.P.); vardas.michail@ucy.ac.cy (M.V.); balaskas.nikolaos@ucy.ac.cy (N.B.); 2 Center for Clinical, Experimental Surgery and Translational Research, Biomedical Research Foundation of the Academy of Athens, 11527 Athens, Greece; nkostom@bioacademy.gr; 3 Laboratory of Experimental Surgery and Surgical Research, Faculty of Medicine, Democritus University of Thrace, 68100 Alexandroupolis, Greece; atsarouc@med.duth.gr; 4 Laboratory of Bioethics, Faculty of Medicine, Democritus University of Thrace, 68100 Alexandroupolis, Greece; 5 Laboratory of Biopharmaceutics-Pharmacokinetics, Department of Pharmacy, School of Health Sciences, National & Kapodistrian University of Athens, 15784 Athens, Greece; valsami@pharm.uoa.gr

**Keywords:** silymarin, silibinin, cardiovascular diseases, diabetes mellitus, hypertension, dyslipidemia

## Abstract

Silibinin/silymarin has been used in herbal medicine for thousands of years and it is well-known for its hepato-protective properties. The present comprehensive literature review aimed to critically summarize the pharmacological properties of silymarin extract and its main ingredient silibinin in relation to classical cardiovascular risk factors (e.g., diabetes mellitus, etc.). We also assessed their potential protective and/or therapeutic application in cardiovascular diseases (CVDs), based on experimental and clinical studies. Pre-clinical studies including in vitro tests or animal models have predominantly implicated the following effects of silymarin and its constituents: (1) antioxidant, (2) hypolipidemic, (3) hypoglycemic, (4) anti-hypertensive and (5) cardioprotective. On the other hand, a direct amelioration of atherosclerosis and endothelial dysfunction after silymarin administration seems weak based on scarce data. In clinical trials, the most important findings are improved (1) glycemic and (2) lipid profiles in patients with type 2 diabetes mellitus and/or hyperlipidemia, while (3) the anti-hypertensive effects of silibinin/silymarin seem very modest. Finally, the changes in clinical endpoints are not robust enough to draw a firm conclusion. There are significant limitations in clinical trial design, including the great variety in doses and cohorts, the underlying conditions, the small sample sizes, the short duration and the absence of pharmacokinetic/pharmacodynamic tests prior to study commitment. More data from well-designed and high-quality pre-clinical and clinical studies are required to firmly establish the clinical efficacy of silibinin/silymarin and its possible therapeutic application in cardiovascular diseases.

## 1. Introduction

Cardiovascular diseases (CVDs) comprise the major mortality and morbidity umbrella of diseases in the modern world [1]. Nowadays, pharmaceutical and non-pharmaceutical interventions modifying cardiovascular risk factors (e.g., diabetes mellitus—DM, hyperlipidemia etc.) predisposing to CVDs are a top priority of scientific research [2,3]. In the context of a healthier lifestyle, herbal medicine has gained an increasing scientific interest from research groups in an effort to develop better therapeutic strategies for primary or secondary prevention of CVDs [4,5].

Many well-designed in vitro and in vivo experimental studies have shown the positive effects of some herb standardized extracts and active ingredients as beneficial supplements in population groups at risk of CVDs. However, there are still many questions to be answered and many studies to be performed to precisely assess the impact of such herbal medicines and/or supplements on humans in order to safely extrapolate experimentally promising results to clinical practice. These questions arise from the heterogeneity of the administered doses due to the different content of the active constituents, the absence of pharmacokinetic (PK) studies and subsequent PK-based dose selection, the heterogeneity of the populations’ characteristics, the possible co-administered pharmaceutical treatment, etc. Accordingly, most of the clinical trials are often characterised by inconclusive findings or have inadequate power, and the results cannot be generalized to the entire population [6,7].

*Silybum marianum* (Milk thistle) is a plant that has been used in herbal medicine for thousands of years. The oldest reported medical use of milk thistle was by Dioscorides [8] recommending the herb as a treatment for serpent bites. Pliny the Elder (Gaius Plinius Secundus—AD 23/24–79) recommended the juice of the plant mixed with honey for “carrying off bile.” Milk thistle was first served as an antidote for liver toxins in the Middle Ages and later by the British herbalist Culpepper to relieve “obstructions of the liver”. In 1898, physicians Felter and Lloyd noted that the herb was helping with the resolution of “liver, spleen and kidney congestion” [8].

Nowadays, silymarin is used as the fruit extract of *S. marianum* and its main constituent silibinin or silybin, constitutes the most active ingredient as confirmed in various studies [8]. This compound belongs to the flavonoid group known as flavonolignans and was classified by the World Health Organization as a potential medication with health-promoting properties in the 1970s. It is derived by organic solvent extraction and represents 1.5–3% of the dry weight of the fruit. Over the last years, apart from the well documented protective effect of silibinin against toxic liver damage [9,10,11,12], the potential anticancer activity of silymarin extract and its main constituent silibinin (e.g., breast, skin, colon, cervix, ovary, prostate, lung, and hepatocellular cancers, among others), has stimulated the interest of the pharmaceutical industry to develop medications with the therapeutic properties of these substances [13]. Furthermore, literature data from experimental in vitro and pre-clinical animal studies also indicate the cardioprotective effects of silymarin extract and its main constituent silibinin, mainly based on its antioxidant, anti-hypertensive, hypolipidemic, hypoglycemic and direct anti-atherogenic properties. In addition, an increasing number of small-scale, observational or randomized, clinical trials have examined the cardiovascular actions of silymarin extract and its main constituent silibinin.

**Figure 1:**
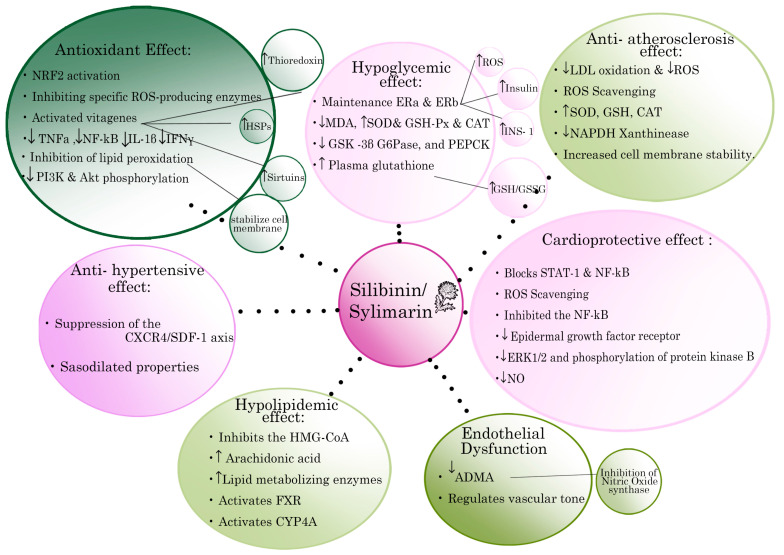
Schematic representation of the most important cardiovascular protective properties of silymarin extract and its main active constituent silibinin and the related possible mechanism of action. Arrows indicate either increase (↑) or decrease (↓) of the respective biomarker expression. Key: IL: interleukin; TNFa: Tumour necrosis factor-a; NF-κB: nuclear factor kappa-light-chain-enhancer of activated B cells; INFγ: Interferon γ; SOD: superoxide dismutase; MDA: malondialdehyde; GPX: Glutathione peroxidase; GSH: Blood Glutathione; GSSG: oxidized glutathione; GSK-3b: glycogen synthase kinase-3b; HMG-CoA: 3-hydroxy-3-methylglutaryl coenzyme A; ERK1/2: extracellular signal-regulated kinase ½; PTP1B, tyrosine phosphatase 1B; NADPH: nicotinamide adenine dinucleotide phosphate; LDL: low density lipoproteins; ROS: Reactive oxygen species; Nrf2: Nuclear factor E2-related factor 2; PI3K: phosphatidylinositol 3-Kinase; Akt: protein kinase A,B; PEPCK: Phosphoenolpyruvate carboxykinase.

The aim of the present comprehensive review was to critically summarize the pharmacological properties of silymarin extract and its main ingredient silibinin, in relation to classical cardiovascular risk factors, such as DM, hyperlipidemia etc., depicted with the related possible mechanisms of action in Figure 1. We also assessed their potential protective and/or therapeutic application in CVDs, based on experimental and clinical studies that are summarized in Table 1 and Table 2 at the end of the results section.

## 2. Literature Search Strategy

We conducted a literature search in the English language for publications in MEDLINE and EMBASE, Web of Science, Cochrane and Google Scholar databases from 1990 to January 2022. The reference lists of the identified articles were checked for any additional relevant articles. The following search terms, in titles and abstracts, including Medical Subject Headings (MeSH) were used: silibinin, silymarin, oxidative stress, antioxidants, inflammation, atherosclerosis, DM, hyperglycemia, hypertension, hyperlipidemia, metabolic syndrome, cardiovascular diseases, and coronary artery disease. Three investigators (N.B., C.P. and M.V.) independently performed the literature search. We included experimental studies, both in vitro and in vivo, and clinical studies. We further limited our literature search by setting the following exclusion criteria: studies with full-text unavailable, published languages other than English, conference abstracts, and interventional arms mixing of silymarin/silibinin and other substances.

Based on abstract and title, we initially found 842 records through database searching of titles and abstracts. After abstracts screening, we removed 188 duplicated studies and another 572 studies as irrelevant. Eighty full-text studies were screened for eligibility. After removing studies with wrong design, irrelevant outcomes, or whose full text was unavailable we ended-up with a total of 29 experimental studies, 4 systematic reviews and meta-analyses of clinical data and 7 clinical studies that were not included in those meta-analyses.

## 3. Results and Discussion

### 3.1. Description, Physicochemical and Biopharmaceutic Properties of Silibinin, Silymarin’s Main Constituent

**Figure 2:**
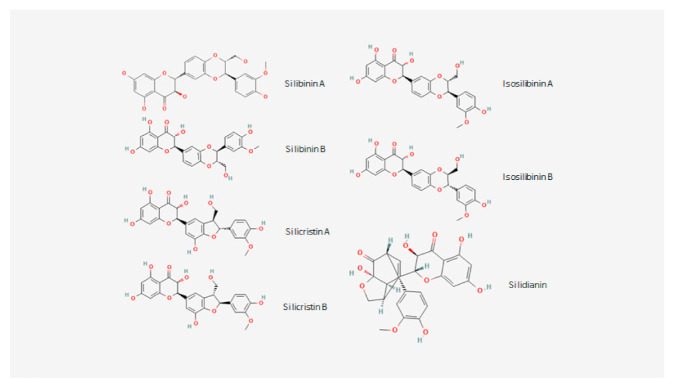
Chemical structures of the main ingredients (flavonoglicans) of Silymarin extract.

Silymarin consists of a mixture of flavonoliglas with the most known being silibinin, isosilibinin, silychristin andsilidianin (Figure 2) [14]. Silibinin is the main active ingredient of silymarin extract, has an IUPAC name of (2R,3R)-3,5,7-trihydroxy-2-[(2R,3R)-3-(4-hydroxy-3-methoxyphenyl)-2-hydroxymethyl)-2,3-dihydro-1,4-benzodioxin-6-yl]-2,3-dihydrochromen-4-one, a molecular formula of C_25_H_22_O_10_, an average mass of 482.4 and a heavy atom count of 35 [15]. Silibinin (also called Silibinine, Silybine, Silybin, Silibininum, Silibinina [14,15]), exists in two diastereomers (Silibinin A and Silibinin B, Figure 1) and is chemically composed of two main units which are linked to one structure by an oxeran ring. The first unit is a taxifolin unit (a flavonoid unit), while the second is a phenylpropanoid unit (a conyferil alcohol) and these determine its behaviour [16,17]. This small and highly functionalized molecule with alternating carbo- and hetero-cycles, is very stable under Bronsted acidic conditions, but its stability is decreased in the presence of Lewis acids or under basic conditions, while prolonged heating over 100 °C or the presence of strong bases may cause changes in its molecular skeleton. Furthermore, Silibinin molecule is resistant to reduction, but it is easily oxidized to 2,3 dehydrasilybin [18].

Silibinin’s structure has 5 hydroxyl groups which are the primary targets of the derivatization process. Three of these hydroxyl groups (5-OH, 7-OH, and 20-OH) possess a phenolic nature, the 5-OH group has a very strong hydrogen bonding to the adjacent oxo group, which is in the conjugation with the aromatic ring and acts as a free electron pair donor to the hydrogen bond with the 5-OH group. Meanwhile, the 7-OH and 20-OH have similar properties, although the C-7 OH group is more reactive than the 20-OH group due to its lower steric hindrance and the presence of a hydrogen bond. The C-23 OH group have properties leading to the esterization or the oxidation of carboxylic groups. The C-3 OH group can easily be oxidized (even with atmospheric oxygen) to a ketone, which is responsible for the creation of a 2,3-dehydrosilybin [18].

Silibinin is poorly soluble in water and in polar protic solvents (EtOH and MeOH), and insoluble in non-polar solvents (chloroform and petroleum ether), but highly soluble in polar aprotic solvents such as DMSO, acetone, DMF, and THF [19]. Silibinin behaves as weak acid in neutral aqueous solutions possessing pKa values of 6.63 for the 5-OH group, 7.7 or 7.95 for the 7-OH group, and 11.0 for the 20-OH group [19,20].

Aqueous solubility of drug substances is one of the main parameters determining oral drug absorption and bioavailability [21]. In the recent study of Kellici et al. [22] the aqueous solubility and the dissolution rate of Silibinin was determined at pH 2.0, 4.5, and 6.8 at 37 °C. The solubility of pure Silibinin was found equal to 0.0033 ± 0.0018 mg/mL at pH 2.0, 0.005 ± 0.0008 mg/mL at pH 4.5, and 0.0023 ± 0.0003 mg/mL at pH 6.8, while its dissolution in the same dissolution media should be considered negligible. These results could be considered in conjunction with the concept of the “Biopharmaceutics Classification System” (BCS) [21], to draw some rough conclusion of Silibinin’s oral bioavailability. BCS has been adopted by both FDA [23] and EMA [24] as described in the relevant regulations, and provides a scientific framework for drug classification into four classes based on their aqueous solubility and gastrointestinal permeability, considering the highest administered dose at a physiologically relevant pH range (pH 1–6.8), as follows: Class I: High Solubility–High Permeability, Class II: Low Solubility–High Permeability, Class III: High Solubility–Low Permeability, Class IV: Low Solubility–Low Permeability. In the same context, Waldamann et al. [25] recently presented a provisional BCS classification strategy for some markers of common herbs used in western medicine, according to the information available from the literature. Based on this classification, the herb Milk thistle (classified using markers Silibinin A and Silibinin B, based on their solubility/permeability properties and considering a dose of 70 mg per marker for a maximum single dose of the commercial product Legalon equal to 140 mg), is suggested to belong to BCS Class III. This classification depicts a substance with low intestinal permeability and high solubility. However, based on the above-mentioned experimental values of silibinin’s aqueous solubility, it should be classified rather as BCS Class IV (low solubility/low permeability) than BCS Class III substance since both silibinin’s solubility and permeability are problematic and limit its intestinal absorption, when a dose of 70 mg is considered.

### 3.2. Antioxidant Actions of Silibinin/Silymarin

The main antioxidant action of silibinin is to maintain an optimal redox balance in the cell by activating multiple enzymatic and non-enzymatic antioxidants, mainly via Nrf2 activation [26]. In this context, it may prevent free radical formation by inhibiting specific ROS-producing enzymes or improving the integrity of mitochondria in stress conditions. As a potent scavenger of reactive oxygen species (ROS) such as hydroxyl and peroxyl anions and hypochlorous acid, silymarin extract, and its main active ingredient silibinin, have shown this action in various systems, such as rat liver microsomes, as well as human platelets, leukocytes, endothelial cells, erythrocytes, and fibroblasts. By scavenging free radicals directly and chelating free Fe and Cu it may affect the microenvironment of the gut, including interactions with bacteria, the latter requiring, however, further investigation [27]. Besides this, in vitro studies have demonstrated that silibinin has the capability to activate vitagenes, responsible for synthesis of protective molecules, such as heat shock proteins (HSPs), thioredoxin and sirtuins and thereby may additionally protect against oxidative stress [27]. On the surface of hepatocytes, silibinin can inhibit organic ion uptake transporters and tumour necrosis factor-alpha (TNF-a) expression, and, in turn, reduce the cellular uptake of xenobiotics, including mushroom poisons [28]. More data have been raised about the protective effect of silibinin on liver toxicity.

Among the proposed mechanisms, silibinin may stabilize cell membrane permeability through inhibition of lipid peroxidation, thereby assisting the liver in maintaining the levels of its own protective antioxidant, glutathione. It also exerts anti-toxic effects against various toxic chemicals, such as carbon tetrachloride, interferon-gamma, interleukin IL-2 and IL-4, by suppressing the pro-inflammatory pathways TNF-a and nuclear factor kappa-light-chain-enhancer of activated B cells (NF-κB). In animal nutrition and disease prevention strategy, silibinin alone, or in combination with other hepato-active compounds (carnitine, betaine, vitamin B12, etc.), might have similar hepatoprotective effects to those described in human nutrition [26].

### 3.3. Silibinin/Silymarin in Atherosclerosis and Ischemia

The effects of silibinin/silymarin on liver disease have been widely studied [29] in the previous decades, but their effects on atherosclerosis have not been as thoroughly explored. Recent studies have given more detailed insight on the possible mechanisms by which flavonoids and particularly silibinin/silymarin exert anti-atherosclerotic actions. Silibinin has been proven to reduce at a dose-dependent manner the atherosclerotic plaque burden in the arteries of hypercholesterolemic rabbits fed with silymarin extract at daily doses of 100 and 200 mg/kg, demonstrating a higher anti-atherosclerotic efficacy of 200 mg/kg/d over 100 mg/kg/d of silymarin [30]. Furthermore, silibinin has been proven to augment the effect of clopidogrel on atherosclerosis [31]. Mice fed with a high fat diet were divided in three groups, Silibinin, Clopidogrel and combined treatment. The mice treated with Silibinin and Clopidogrel showed reduced aortic lesion, inflammation and endothelial dysfunction. Also, the anti-thrombotic effect of clopidogrel has also been augmented in the mice treated with both substances. Another animal study examined the dose-dependent effects of silymarin on atherosclerosis development in New Zealand rabbits receiving a high-fat diet. However, the extrapolation of those results to humans should be done with caution, requiring well-designed and properly conducted clinical studies.

Regarding the underlying mechanisms, silymarin/silibinin may lower LDL oxidation, which is an intermediate step of atherosclerosis development [32]. The contributory role of excessive formation of Radical Oxygen Species (ROS) to atherogenesis has been well-studied. Their accumulation in the vascular wall promotes LDL oxidation and this in turn promoted atherosclerotic plaque formation, especially at early stage [32]. Therefore, the athero-protective mechanisms of silymarin/silibinin involves significant antioxidant properties and reduced LDL oxidation [27]. Although the atheroprotective impact of other antioxidants, beyond silibinin, has been supported by a plethora of experimental data [33], their clinical effectiveness remains a matter of debate. The low bioavailability of antioxidants has explained the discordance between experimental and human results restricting significantly their implementation in atherosclerotic diseases. While Silibinin seems to be a highly effective antioxidant, among other things, its properties require further investigation [27]. In this context, silibinin/silymarin may improve vascular function. Experiments using rat models have proved a significant amelioration of pulmonary vascular dysfunction after receiving 250 mg/kg/day of silymarin for eight days [34] and retrieval of normal endothelium and vascular elasticity after lung ischemia reperfusion injury on an ex vivo model of isolated rat aortas [35]. Furthermore, silibinin (20 mg/Kg i.p. daily) alleviates endothelial dysfunction by decreasing the asymmetric dimethylarginine (ADMA) levels in the aorta and plasma, which reverses the inhibition of Nitric Oxide synthase [36] and so increases NO availability [37].

Along with atherogenesis, myocardial infarction has been studied in rats. To prevent myocardial necrosis, prompt reperfusion is the gold standard target in myocardial infarction, at the expense of reperfusion injury characterized by a paradoxical rise in cell death. The underlying mechanisms of reperfusion injury involve an increase of mitochondria permeability transition and the myocardial cellular apoptosis shortly after reperfusion followed by ROS excretion [38]. Such cardioprotective effects of silibinin have been very recently examined in mice undergoing Left Anterior Descending Artery (LAD) ligation which were randomized to either pre-treatment with silibinin, 100 mg/kg for seven days, prior to myocardial infarction or remained untreated [39]. In the silibinin-treated group cardiac dysfunction induced by Infraction/Reperfusion (I/R) injury was significantly ameliorated compared to the sham group. This was implicated by the significant increase of the left ventricular ejection fraction (LVEF) and the lowered levels of B-type natriuretic peptides and cardiac troponin in the former group. In another animal study, the infarct size was reduced in a dose-dependent manner in the silymarin treated group, (silymarin 100, 250, 500 mg/kg) [40]. Taken altogether, these preliminary data are strong evidence of the potential athero- and cardio-protective properties of silibinin.

### 3.4. Silibinin/Silymarin and Metabolic Syndrome (MS)

In general, Silibinin/Silymarin exerts multiple beneficial effects on metabolic syndrome (MS) aspects, via a variety of mechanisms and actions [41]. According to the International Diabetes Federation, diagnostic criteria for the MS include [42]: elevated levels of triglycerides and fasting blood glucose (FBG), reduced levels of high-density lipoprotein (HDL), elevated blood pressure and increased waist circumference [43]. There are not many in-vivo and in-vitro studies examining the possible positive effect of the flavonoids, including silymarin ingredients. Summarizing the results of those studies, we can reach the conclusion that silibinin may favourably change cardiovascular risk factors clustered under MS definition. In particular, silymarin increases the HDL levels, but its impact on total cholesterol level is controversial [44,45]. According to a few studies, it may significantly decrease blood pressure, body weight, FBG and triglycerides [44,45,46,47,48,49]. In the following sections we have analysed the impact of silibinin/sylimarin on each cardiovascular disease (CVD) risk factor separately. In case of MS as a cluster of those factors, sylimarin seems to decrease the total oxidative stress (30, 100, 300 mg/kg) [46,50], particularly targeting oxidant secretion by the liver [43]. Moreover, the hepatoprotective properties of silibinin may favourably modulate a number of metabolic parameters. In particular, it can suppress the production and release of inflammatory mediators from the liver which significantly contribute to CVD development and manifestation [47,48].

Finally, an animal study has been conducted in order to ascertain silymarin’s possible action on hyperuricemia [51]. The latter is the most important risk factor for the development of gout [52] and is, in parallel, a risk factor for MS and CVD, through vascular inflammation and oxidative stress [53]. It usually accompanies diseases of vascular endothelial dysfunction. Anti-hyperuricemic therapy targets uric acid production via a xanthine oxidase inhibitor (XOD-inhibitor), so-called allopurinol. It blocks xanthine oxidase (XOD), the enzyme catalysing the oxidation of hypoxanthine to xanthine and eventually to uric acid. With the exception of the silibinin, some flavonoids have a similar hypouricemic action, through the inhibition of XOD, however hyperuricemia seems to be unaffected by silibinin administration.

### 3.5. Silibinin/Silymarin and Diabetes Mellitus

DM constitutes an epidemic disease in western societies with a high burden on healthcare systems worldwide. In 2019, DM was the ninth leading cause of death with an estimated 1.5 million deaths directly linked to DM [54]. The rapidly growing incidence of DM has increased the demand for new effective medications and diet modification. In this context, an increasing number of studies [55] have examined the antidiabetic properties of silymarin, and its major component silibinin, as a dietary supplementation.

Animal studies in diabetic rats have also shown the glucose-lowering actions of silymarin at daily doses ranging from 60 mg/kg to 300 mg/kg [56,57,58,59,60]. In addition to FBG reduction, in vivo studies have shown improved glucose homeostasis [61] and higher insulin sensitivity in adipose tissue after sylimarin administration (40 mg/100 g) [48]. In some of these, the effects derived from silymarin-induced elevation of serum insulin levels [56,62]. The investigators suggested increased pancreatic β-cells number, up-regulation of their viability and improvement in their function in vivo and in vitro (high glucose-palmitate/BSA-treated β-cells) as well [56]. In contrast, two studies failed to show an improvement in beta cells function (silibinin 50 mg/kg/day & 100 mg/kg/day) [63] or hyperglycemia (silymarin 15 mg/kg/day & 30 mg/kg/day) [64] in diabetic animals in a dose dependent manner. However, the latter study showed a significant reduction in body weight and serum insulin levels [64]. Alloxan has been used in diabetic animal models due to its destroying effects on pancreatic β-cells [58]. Silymarin can significantly increase the pancreatic and plasma glutathione levels, which in turn enhances the GSH/GSSG ratio and prevents pancreatic lipid peroxidation and the consequent hyperglycemia induced by alloxan in rats [58]. Moreover, silymarin (200 mg/kg/day) augments the activity of pancreatic antioxidant enzymes restoring the alloxan-induced toxicity on β-pancreatic cells leading to normalization of blood glucose levels [65]. The pancreatic β-cells damage can be inhibited by activating ERα-dependent Nrf2-antioxidative signalling pathways [56] and reducing the production of free radicals [58]. The ERa receptors may also trigger insulin synthesis in pancreatic β-cells and thereby maintain glucose homeostasis [51]. Although sylimarin’s effect on insulin tolerance in diabetic animals is not well tested, it has been reported to ameliorate insulin tolerance in obese mice without T2D after silymarin treatment [61].

In addition to oxidative stress suppression, silymarin has been proved to decrease pro-inflammatory cytokines, like TNF-α and IL-1β (Interleukin-1β) in diabetic rats [56]. A lower expression of TNFa and IFNγ (Interferone-γ) has been found in diabetic silymarin-treated zebrafish [66]. In vitro studies have reported reduced TNFα/IL-1β mRNA expression in insulin secreting (INS) cells pre-treated with silymarin. Counterbalancing the decreased protein expression of Phosphoinositide 3-kinases (PI3K) and Protein kinase B (Akt) phosphorylation in pancreatic cells and pre-treatment with silymarin of the cells under TNFα/IL-1β treatment increased the expression of PI3K and Akt phosphorylation [62].

Regarding the protective effects of silymarin on diabetic complications Miranda et al. (2020) [63] demonstrated reduced hepatic and pancreatic protein damage, creatinine levels, and food and water intake after silymarin administration (50 and 100 mg/kg body weight/d for 30 consecutive days). Concomitantly, silymarin prevented the loss of bodyweight and dehydration, likely by reducing daily urine volume, which consequently led to less water intake [63]. Similarly, silymarin exerted a nephron-protective action in rats with streptozotocin- and nicotinamide-induced type 2 diabetic nephropathy [57]. Silymarin also contains considerable number of phenolic compounds which may play an important role in stabilizing lipid peroxidation due to their intrinsic reducing capacity [67]. Moreover, declined levels of cholesterol and triglycerides have been observed in diabetic rats after treatment with silymarin. These findings might suggest a hypocholesterolemic activity of silymarin in diabetic animals [59].

### 3.6. Silibinin/Silymarin and Hyperlipidemia

The hypolipidemic effects of silymarin have also been the focus of various studies. In rats with hyperlipidemia developed after either a high-fat diet or with N-nitrosodiethylamine (NDEA) silymarin may significantly decrease total cholesterol triglycerides [68], VLDL and LDL [61,69]. Moreover, silymarin seems to increase HDL concentrations [44]. It is important to mention that an in vivo study showed that N-nitrosodiethylamine (NDEA) induced increased levels of cholesterol and triglycerides in animals with subsequent decrease in the levels of phospholipids and free fatty acids in serum. Rats that were treated with both NDEA and sylimarin (1000 mg/L/day for 16 weeks) showed a significant decrease in the levels of cholesterol and triglycerides with subsequent increase in the levels of phospholipids and free fatty acids compared with the NDEA group [68]. Silymarin exhibited its potent hypolipidemic effect by increasing the levels of lipid metabolizing enzymes in the liver and decreasing cholesterol/phospholipid ratio both in the serum and liver [68]. It also inhibits 3-hydroxy-3-methylglutaryl coenzyme A (HMG-CoA) reductase activity, which is the rate limiting enzyme in cholesterol biosynthesis [68]. In cases of triglycerides (TGs) decline, this is mainly attributed to triggered triglycerides metabolism through CYP4A [44,45]. Notably, silymarin increases the level of arachidonic acid [68] and activates the Farnesyl X receptor (FXR) transactivity in a dose-dependent manner [61]. FXR has been shown to play an essential role in controlling normal lipid and glucose metabolism by regulating the expression of a series of downstream target genes [61].

### 3.7. Silibinin/Silymarin and Hypertension

There is a very small number of studies regarding the impact of silibinin/silymarin on hypertension. Those studies mainly assess hypertension in the context of liver disease or in combined administration with other compounds (e.g., tetrandrine) [70]. An old animal study conducted in hypertensive rats [70] investigated the effects of silibinin (300 mg/kd/day) on blood pressure, and ventricular hypertrophy, from one side, and infarct size and death rate after coronary artery ligation, on the other. The authors concluded that silibinin reduced blood pressure, left ventricular hypertrophy, arrhythmias, and mortality in those rat models. Rat death rates decreased after oral consumption of silibinin and lowered blood pressure with the same efficacy as the drug tetrandrine, presumably due to its antioxidant activity [27]. The vasodilated properties of silymarin have been implicated in two more studies using rat models [71,72]. Silymarin administration (200 mg/kg/day for 5 weeks) significantly ameliorated pulmonary artery hypertension (PAH) at early stage before it became a severe and irreversible condition. The underlying mechanism may involve the suppression of a chemokine/receptor axis, CXCR4/SDF-1 axis (CXCR4 is a chemokine receptor and SDF-1, stromal cell derived factor, is its ligand), which may delay pulmonary arteriolar occlusion and pulmonary vascular remodelling, and thus can ameliorate pulmonary arterial hypertension (PAH) [73,74,75].

Up-to-date data about the hypotensive activity of silibinin/silymarin are very scarce, and it is clear that more studies are required to unravel the underlying mechanisms of silibinin/silymarin’s effect on arterial systemic hypertension.

### 3.8. Silibinin/Silymarin and Cardiomyopathies

In recent research, heart failure, cardiomyopathies and cardiotoxicity are therapeutical targets of silymarin research with encouraging results. More specifically, silymarin treatment of diabetic mice (100 mg/kg) has shown it ameliorates diabetic cardiomyopathy [76]. This study proved that silymarin treatment decreased cardiac fibrosis and collagen deposition. Moreover, the echocardiographic evaluation of silymarin-treated mice showed attenuated cardiac dysfunction. Silibinin has also been proven to reduce the hypertrophic response in H9c2 rat embryonic heart cells induced by phenylephrine possibly by antioxidant mechanisms mediated by extracellular signal-regulated kinase ½-mitogen activated protein kinases (ERK1/2 MAPKS) [77]. TPM1 encodes the actin binding protein tropomyosin 1 which plays a role in striated muscle contraction. Myosin Light Chain (2MYL-2) has been proven to play a crucial role in the normal development of ventricular cardiac myocyte structure and function [78]. Silibinin has been proven to increase the gene expression of these two genes [79]. The study that described this effect involved obese mice divided in two groups (High fat diet and High fat diet plus silibinin). The group that was treated with silibinin (54 mg/kg/day for 4 weeks) showed the greater expression of the above genes. This study regarded the presence of these genes in the area of diabetic cardiomyopathy. Both proteins involving TPM1 [80] and MYL2 [81] in dilated and hypertrophic cardiomyopathy are strongly supported as well. Potentially this could be a future role of silibinin for the development of newer therapeutic agents, targeting the genes responsible for several types of cardiomyopathies. However, the proposed mechanism requires further investigation since the role of TPM1 and MYL2 in cardiomyopathies has not been adequately explored.

Cardiotoxicity is a well-known side effect of chemotherapy associated with major morbidity in cancer patients. Silymarin may provide protection against those adverse effects [82]. Doxorubicin, a widely used chemotherapeutic agent, is commonly accompanied by cardiotoxicity. In vitro pretreatment of rat cardiomyocytes with silymarin decreased the cardiotoxicity of doxorubicin at a dose-dependent manner (25–100 μm) [83,84], while silymarin (silibinin 60 mg/kg, orally) may protect the heart of doxorubicin-treated rats [85]. Another in vitro study of doxorubicin showed reduced serum markers of Lactate dehydrogenase (LDH), creatine kinase myocardial band (CK-MB) and cTroponin I after silymarin administration (50 mg/kg/day), implicating less myocardial damage possibly through its anti-oxidative properties [86]. Cisplatin is also an established chemotherapeutic drug, and the cardioprotective properties of silymarin have been described [87]. In particular, silymarin administration reduced serum level biomarkers of myocardial damage, such as LDH, creatinine kinase isoenzymes and cardiac troponin I, in an albino rat study. Adriamycin (ADR) is another potent anti-cancer drug that has limited use due its adverse effects. An in vivo study of ADR-induced cardiotoxicity in albino rats proved lower LDH and creatine phosphokinase (CPK) plasma levels in the silymarin pre-treated group (50 mg/kg for 30 days) compared with the control group [88]. Light micrograph of rat heart specimens showed milder myocardial injury (milder interstitial edema, vascular congestion and subendocardial cell infiltration) in the group of silymarin treatment preceding ADR administration. Therefore, the cardioprotective mechanisms of silibinin/silymarin against cardiotoxic agents warrant further investigation. Potentially, silymarin could be an essential part of novel strategies in cardiomyopathies.

### 3.9. Silibinin/Silymarin and Cardiovascular Prevention Based on Clinical Studies

To our knowledge, there is a limited number of clinical trials and meta-analyses concerning the direct or indirect cardioprotective actions of Silymarin and its main component, Silibinin. In particular, four systematic reviews have analysed randomized clinical trials (RCTs) investigating the effects of Silibinin/Silymarin on patients with type 2 diabetes mellitus (T2DM) and/or metabolic disorders. Both are known as major cardiovascular risk factors and their modification is of clinical importance.

The first published meta-analysis [55] included five double and triple blinded RCTs, enrolling 270 patients. The systematic silymarin administration in patients with T2DM improved glycemic control, by reducing FBG and Glycated haemoglobin (HbA1c) modestly. There was no effect of silymarin on lipid profile, and an indefinite effect on chronic kidney disease progression. Additionally, it did not reduce the risk of cardiovascular mortality (RR 1.11; 95% CI 0.92–1.35), but intensive treatment appeared to reduce the risk for nonfatal myocardial infarction (RR 0.85, 95% CI 0.76–0.95, and *p* = 0.004) although this was not confirmed in trial-sequential analysis. The authors commented that the high heterogeneity of the studies (small number of studies with small sample sizes, various silymarin products and dosages and short-term follow-up) were major limitations of their meta-analysis.

In contrast, a slightly larger meta-analysis published in 2018 [89], including 7 RCTs (370 patients with T2DM), revealed the beneficial effects of silymarin supplementation on FBG, HbA1c, Insulin, LDL-Cholesterol, malondialdehyde (MDA) and HDL-Cholesterol levels. Non-significant effects were detected for total cholesterol and triglycerides. Again, the validity of the results was limited due to the high heterogeneity leading to insufficient evidence. A third meta-analysis published in 2019 [90] including 10 RCTs (620 adults with dyslipidemia), focused on silymarin’s effects on lipid profile, while in half of the studies there were concomitant therapies. Silymarin supplementation significantly ameliorated all lipid parameters, but methodological issues, such as the wide variation in the duration (45 days to 12 months) and the dosage (280 mg to 2100 mg/day) of supplementation, weakened the strength of evidence. Additionally, Iran was the origin of 6 out of 10 trials. Finally, the most recent meta-analysis targeted a wider spectrum of patients with glucose/metabolic dysfunction [91]. It analysed 16 published clinical studies, 15 of them were RCTs, enrolling 1358 patients assessing the therapeutic effects of silymarin. It concluded that silymarin supplementation significantly reduced the levels of FBG, HbA1c, insulin resistance (HOMA-IR), and CRP, while it ameliorated all lipid parameters in agreement to previous meta-analysis. Silymarin did not affect markers of liver, kidney and muscular function (e.g., ALT, AST, creatinine, creatinine phosphokinase), and did not increase the complication rate compared to control groups. The investigators hypothesized an anti-inflammatory impact of silymarin in addition to classical cardiovascular risk factors modification.

In addition to the clinical trials included in the aforementioned meta-analyses, other studies have examined the effects of Silibinin/Silymarin mostly on T2DM and hyperlipidemia and to a lesser extent on renal function and arterial stiffness. The majority of these were randomized, double or triple-blinded, placebo-controlled clinical trials [92,93,94,95,96]. Milk thistle extract (*Silybum marinarum extract*, *Silymarin*) or silibinin, was administered alone or in combination with vitamins (D, E) and other herbal compounds. Notably, all those clinical trials demonstrated improved glycemic indices, insulin sensitivity and lipid profile in T2DM patients after Silibinin/Silymarin supplementation. More studies using other target populations: cirrhotic diabetic patients [97,98], non-alcoholic fatty liver disease patients (NAFLD) [99,100], alcoholic liver disease patients [101], and hypertensive diabetic patients [102] documented similar favourable outcomes in metabolic parameters. Alkuraishy HM et al. [103] reported that silymarin can be used alone effectively and safely in the treatment of hyperlipidemia of different aetiology.

Silymarin was tested using the well-known procedure of coronary artery bypass graft (CABG). A number of 140 patients undergoing CABG under cardiopulmonary bypass were treated pre-operatively with silymarin. A significant decrease in values such as white blood cell count, LDL, serum creatinine and HbA1c in diabetic patients was observed [104]. No relation with clinical outcomes was mentioned.

Some other studies examined the effect of silibinin on patients with end-stage renal disease (ESRD) on haemodialysis or peritoneal dialysis. Those studies showed that silymarin alone or in combination with other supplements (vitamin E) can reduce MDA levels in plasma, increase the level of red blood cells and increase haemoglobin levels [105,106]. However, those studies were of small sample size. Finally, two RCTs [107,108] searched the complementary effects of silymarin with renin-angiotensin system inhibitors on proteinuria, in T2DM patients with diabetic nephropathy. Both studies failed to show any significant impact of silymarin on survival and renal progression in those patients, with the exception of the subgroup of patients with lower estimated glomerular filtration rate (eGFR) or higher proteinuria levels. Given that renal function is directly related to the cardiovascular system, this may be another indirect index for silibinin’s possible cardioprotective role [109].

To our knowledge, there is a limited number of clinical trials and meta-analyses concerning the direct or indirect cardioprotective actions of silymarin and its main active component, silibinin (Table 1). In particular, four systematic reviews have analysed randomized clinical trials (RCTs) investigating the effects of Silibinin/Silymarin on patients with T2DM and/or metabolic disorders [55,89,90,91]. Both are known as major cardiovascular risk factors and their modification is of clinical importance.

**Table 1:** Published meta-analysis studies of randomized clinical trials investigating the impact of silibinin/silymarin on classical cardiovascular risk factors.

Reference	Total Studies & Cohorts–Dose Range	Study Objectives	Outcomes
Voroneanu, et al., 2016 [55]	5 RCTs, 270 pts, T2DM Silymarin extract Daily dose 200 and 600 mg	Metabolic parameters, CKD progression, cardiovascular mortality and morbidity	↓ glycemic indices ↔ lipid profile, indefinite effect on CKD, ↔ cardiovascular mortality, ↓ risk for nonfatal MI (only in intensive treatment)
Hadi, et al., 2018 [89]	7 RCTs, 370 pts, T2DM Silymarin extract Daily dose 200, 420 and 600 mg	Metabolic parameters	↓ FBG, ↓ HbA1c, ↓ fasting Insulin, ↓ LDL-C, ↑ HDL-C, ↔ TChol, ↔ TG, ↓ MDA
Mohammadi, et al., 2018 [90]	10 RCTs, 620 pts, dyslipidemia Silymarin extract Daily dose range 280 to 2100 mg	Metabolic parameters	↓ LDL-C, ↓ TG, ↓ TChol, ↑ HDL-C
Xiao, et al., 2020 [91]	15 RCTs & 1 prospective study, 1358 pts, T2DM and/or dyslipidemia Silymarin extract Daily dose range 105 to 1000 mg	Metabolic parameters	↓ FBG, ↓ HbA1c, ↓ HOMA-IR, ↔ FBI, ↓ LDL-C, ↓ TG, ↓ TChol, ↑ HDL-C, ↓ CRP, ↔ MDA, ↔ ALT, ↔ AST, ↔ CPK, ↔ creatinine, ↔ Phosphokinase

Key: ALT, Alanine transaminase; AST, Aspartate transaminase; CKD, chronic kidney disease; FBG, Fasting blood glucose; FBI, fasting blood insulin; HbA1c, glycosylated haemoglobin; HDL-C, High-density lipoprotein-Cholesterol; HOMA-IR, Homeostatic Model Assessment for Insulin Resistance; CRP, C-reactive protein; LDL-C, Low-density lipoprotein-Cholesterol; MI, myocardial infarction; MDA, Malondialdehyde; pts, patients; RCT, randomize controlled trials; T2DM, type 2 diabetes mellitus; TG, triclycerides; ↓, decrease; ↑, increase; ↔, significant change.

**Table 2:** Randomized Clinical trials not included in the published meta-analysis studies, investigating the impact of silibinin/silymarin on classical cardiovascular risk factors.

Reference	Cohorts	Study Design	Outcomes-Variables
Ebrahimpour Koujan, et al., 2015 [92]	40 pts, T2DM	RCT; Intervention group (20 pts): 140 mg Silymarin orally, 3 times/day, for 45 d Control group (20 pts): placebo	↑ SOD, ↑ GPX, ↑ TAC, ↓ hs-CRP, ↓ MDA
Ebrahimpour-Koujan, et al., 2018 [93]	40 pts, T2DM, dyslipidemia	RCT; Intervention group (20 pts): 140 mg Silymarin orally, 3 times/day, for 45 d Control group (20 pts): placebo	↓ FBS, ↓ insulin, ↓ HOMA-IR, ↑ QUICKI, ↓ TG, ↓ TChol, ↑ HDL-C, ↓ LDL-C
Hussain SA, et al., 2007 [94]	51 pts, T2DM	RCT; Group A (18 pts): 200 mg/day Silymarin orally + 10 mg/day oral glibenclamide for 120 d Group B (20 pts): placebo + 10 mg/d oral glibenclamide, for 120 d Group C (control) (21 pts): 10 mg/d glibenclamide	↓ FBG, ↓ HbA1c, ↓ insulin, ↓ LDL-C, ↓ TG, ↓ TChol, ↓ SGOT, ↓ SGPT, ↓ weight, ↓ SBP ↓ DBP
Khalili, et al., 2017 [95]	60 pts, T2DM	RCT; Intervention group (30 pts): 200 mg Silymarin orally, 3 times/day, for 90 d Control group (30 pts): placebo	↓ FBG, ↓ HbA1c, ↓ TG, ↓ TChol, ↔ LDL-C, ↔ HDL-C, ↔ SBP, ↔ DBP, ↔ AST, ↔ ALT, ↔ ALP, ↔ BUN, ↔ Creatinine
Huseini HF, et al., 2006 [96]	51 pts, T2DM	RCT; Intervention group (25 pts): 200 mg silymarin orally 3 times/day, for 120 d Control group (26 pts): placebo	↓ FBG, ↓ HbA1c, ↓ BMI
Velussi M., et al., 1997 [97]	60 pts, insulin-treated DM, liver cirrhosis	Randomized, open, controlled study; Intervention group (30 pts): standard therapy + 200 mg Silymarin orally, 3 times/day, for 360 d Control group (30 pts): standard therapy	↓ FBG, ↓ glucosuria, ↓ HbA1c, ↓ insulin, ↓ MDA, ↓ C-peptide, ↔ γGT, ↔ ALP ↔ creatinine, ↔ bilirubin, ↔ microalbuminuria, ↓ AST, ↓ ALT, ↓ TChol, ↑ HDL-C, ↑ TG
Federico A, et al., 2019 [99]	90 pts with NAFLD and 60 healthy participants	Prospective study; Intervention group (NAFLD, 60 pts): 1 capsule 2 times/day, for 180 d [capsules: silybin-phospholipid complex (303 mg) of, vitamin D (10 mg), vitamin E (15 mg)]. Control group (NAFLD, 30 pts): no drug Healthy group (60 pts): no drug	↔ BMI, ↔ weight, ↓ ALT, ↓ γGT, ↔AST, ↓ insulin, ↓ HOMA-IR, ↑ vitamin D, ↓ degree of steatosis ↔ FBG, ↔ TG, ↔ TChol, ↔ LDL-C, ↔ Ferritin
Cerletti C, et al., 2020 [100]	RCT, 126 pts, NAFLD	RCT; Intervention group (62 pts): 2 capsules, once a day, for 90 d [capsules: mixture of active ingredients, 70% DHA (250 mg), phosphatidylcholine (150 mg), silymarin (75 mg), choline bitartrate (35 mg), curcumin (35 mg) and D-α-tocopherol (10 mg)] Control group (64 pts): placebo	↓ AST, ↑ HDL, ↑ LDL, ↑ TChol, ↑ FBG, ↔ weight, ↔ BMI, ↔ waist circumference
Sciacqua A, et al., 2019 [102]	50 pts, Hypertension	Pilot, single arm, interventional, longitudinal study. Dose: 3 g Silibinin, twice a day (Silibinin conjugated to vit E and phosphatidylcholine–oral solution)	↓ TChol, ↓ TG, ↑ HDL-C, ↓insulin, ↓ FBG, ↓ HOMA-IR, ↑IGF-1, ↑ eGFR, ↓ CRP, ↓ UA, ↓liver enzymes, ↓ SBP, ↓ Pulmonary pressure
Alkuraishy, et al., 2012 [103]	20 pts, Dyslipidemia	RCT; Intervention group (10 pts): 600 mg silymarin orally, once/day, for 14 d Control group (10 pts): placebo	↓ TChol, ↓ TG, ↑ HDL-C, ↓ LDL-C, ↓ VLDL
Altaei T, et al., 2012 [104]	102, CABG	Prospective study; Intervention group (50 pts): 140 mg silymarin orally, 3 times/day, 3 d before surgery Control group (52 pts): no drug	↓ cytokine concentrations (IL-6, IL-1a, TNF-a), ↓ CRP, ↑ GSH, ↑ TEAC, ↓ MDA
Roozbeh J, et al., 2011 [105]	80, ESRD, DM, Hypertension	Prospective study; Group 1 (20 pts): 140 mg silymarin, orally, 3 times/day, for 90 d Group 2 (20 pts): vitamin E 400 IU/day, for 90 dGroup 3 (20 pts): 140 mg silymarin, orally, 3 times/day + vit E 400 IU/day, for 90 d Group 4, control (20 pts): no drug	↑ GPX, ↓ MDA, ↑ mean hemoglobin (all three treatment groups VS control)
Firuzi O, et al., 2016 [106]	60, ESRD	RCT; Intervention group (28 pts): 140 mg silymarin orally, 4 times/day, for 60 d Control group (22 pts): placebo	↓ FRAP, ↑ Hemoglobin, ↑ serum albumin, ↔ creatinine, ↔ iPF2a
Voroneanu L, et al., 2017 [107]	102, T2DM, Proteinuria, (ischemic heart disease)	RCT; Intervention group (51 pts): 150 mg silymarin, orally, 3 times/day, for 2 years (720 d) Control group (51 pts): placebo	↔ mortality ↔ progression of CKD indefinite effect on eGFR and proteinuria

Key: ALP, Alkaline phosphatase; ALT, Alanine transaminase; AST, Aspartate transaminase; BMI, Body mass index; BUN, blood urea nitrogen; d, days; DBP, diastolic blood pressure; DM, diabetes mellitus; eGFR, estimated Glomerular filtration rate; ESRD, End-stage renal disease; FBG, Free blood glucose; FRAP, Ferric Reducing Antioxidant Power; GPX, Glutathione peroxidase; GSH, Blood Glutathione; HbA1c, Haemoglobin A1c; HDL-C, High-density lipoprotein Cholesterol; HOMA-IR, Homeostatic Model Assessment for Insulin Resistance; hs-CRP, high-sensitivity C-reactive protein; IGF-1, Insulin-like growth factor 1; iPF2a, 8-iso-prostaglandin F2a; LDL-C, Low-density lipoprotein Cholesterol; MDA, Malondialdehyde; NAFLD, Non-Alcoholic Fatty Liver Disease; pts, patients; QUICKI, quantitative insulin sensitivity check index; RCT, randomized control trial; SBP, systolic blood pressure; SOD, superoxide dismutase; TAC, total antioxidant capacity; TEAC, Plasma trolox equivalent antioxidant capacity; TChol, Total cholesterol; TG, Triglycerides; TGFβ, Transforming growth factor beta; TNF-a, tumour necrosis factor-a; UA, uric acid; UACR, Urinary albumin-creatinine ratio; VLDL-C, Very-low-density lipoprotein cholesterol; γGT, gamma-glutamyl transpeptidase; ↓, decrease; ↑, increase; ↔, significant change.

## 4. Study Limitations

There are several drawbacks before considering the net results of silibinin/silymarin on cardiovascular risk factors and diseases. The published data are quite promising; however, the mechanistic explanations are derived from mostly in vitro rather than in vivo studies. Despite the growing number of studies, the underlying mechanisms of silibinin/silymarin actions remain unclear or somehow speculative and require further investigation using the appropriate animal models. The aforementioned RCTS [92,93,94,95,96,97,99,100,102,103,104,105,106,107] are of small sample size, the studied interventions administering silymarin as a food supplement are inhomogeneous with a great variety in dosage, duration of treatment and patients’ characteristics. Moreover, they have targeted mostly biomarkers and not clinical outcomes, since they have examined silymarin as a measure of primary cardiovascular prevention enrolling patients with predisposition for CVD (e.g., T2DM). Despite the existing meta-analyses [55,89,90,91], the included RCTS were underpowered to assess the clinical impact of silymarin for secondary prevention. Accordingly, based on the existing knowledge, although a modest improvement in glycemic and lipid profile has been documented, products of silibinin/silymarin seems hard to pass the evaluation in FDA or EMA for drug approval, and so can be only applied as healthy food supplement. Last but not least, apart from the very old study of Weyhenmeyer et al. [110] on dose-linearity of the pharmacokinetics of silibinin, the study of Wen et al. [111] on pharmacokinetics and metabolic profile of silibinin, and the recent study Sornsuvit et al. [112] on the bioavailability and pharmacokinetics of silymarin SMEDDS, we did not identify any other pharmacokinetic/pharmacodynamic trial before any clinical application of silymarin as a dietary supplement which significantly compromises the validity and reproducibility of clinical studies.

## 5. Conclusions

Silymarin and its major component silibinin are well-known for their hepato-protective actions. There is growing evidence supporting their significant cardiovascular protective properties, such as antioxidant, anti-inflammatory, hypotensive, glucose and lipid-lowering in pre-clinical studies. A limited number of clinical studies have documented a modest improvement in glycemic and lipid profile, but there are some important limitations leading to inconsistent results. Additional, larger, properly designed clinical trials, in specific conditions, with standard dosages and properly designed pharmacokinetic/pharmacodynamic studies are required to test its value as food supplement to conventional drug therapies.

## Data Availability

Not applicable.
